# Effectiveness of Vestibular Training for Balance and Dizziness Rehabilitation in People with Multiple Sclerosis: A Systematic Review and Meta-Analysis

**DOI:** 10.3390/jcm9020590

**Published:** 2020-02-21

**Authors:** Cristina García-Muñoz, María-Dolores Cortés-Vega, Alberto Marcos Heredia-Rizo, Rocío Martín-Valero, María-Isabel García-Bernal, María Jesús Casuso-Holgado

**Affiliations:** 1 Department of Physiotherapy, University School Fco. Maldonado, Avd. de los Cipreses s/n, 41640 Osuna, Spain; cristinagm@euosuna.org; 2 Department of Physiotherapy, Faculty of Nursing, Physiotherapy and Podiatry, University of Seville, C/Avicena s/n, 41009 Seville, Spain; amheredia@us.es (A.M.H.-R.); ibernal@us.es (M.-I.G.-B.); mcasuso@us.es (M.J.C.-H.); 3 Department of Physiotherapy, Faculty of Health Sciences, University of Malaga, Arquitecto Francisco Peñalosa 3, Ampliación de Campus de Teatinos, 29071 Malaga, Spain; rovalemas@uma.es

**Keywords:** multiple sclerosis, vestibular diseases, postural balance, dizziness, fatigue, physical therapy modalities

## Abstract

Postural instability and dizziness are commonly observed in people with multiple sclerosis (PwMS). The aim of this systematic review was to evaluate the evidence for the use of vestibular rehabilitation, in comparison with other exercise interventions or no intervention, to treat balance impairments and dizziness in PwMS. An electronic search was conducted by two independent reviewers in the following databases: MEDLINE (Pubmed), Scopus, the Physiotherapy Evidence Database (PEDro), Web of Science (WOS), Lilacs, CINHAL and the Cochrane Database of Systematic Reviews (CDSR). A quality assessment was performed using the PEDro scale and the Cochrane Risk of Bias Tool. When possible, the data were pooled in a meta-analysis (95%CI). This systematic review followed the PRISMA guideline statement and was registered in the PROSPERO database (CRD42019134230). Seven studies were included, with a total of 321 participants analysed. Compared with no intervention, vestibular rehabilitation was more effective for balance development (SMD = 2.12; 95% CI = 0.49, 3.75; *p* = 0.01; I^2^ = 89%) and dizziness symptoms improvement (SMD = −17.43; 95% CI = −29.99, −4.87; *p*= 0.007; I^2^= 66%). Compared with other exercise interventions, improvements in favour of the experimental group were observed, but statistical significance for the differences between groups was not reached.

## 1. Introduction

Multiple sclerosis is an autoimmune disease of the central nervous system that affects approximately 2.5 million people in the world at present. It is a complex disease characterised by a wide variety of symptoms [1].Among these symptoms, dizziness, including postural intolerance, has been reported to affect 49–59% of people with multiple sclerosis (PwMS) [2], and balance disorders are observed in 75–82% of mild to moderate disable subjects [3]. Sensory impairments in the visual, vestibular and proprioceptive pathways have been associated with these symptoms [3,4]. Furthermore, a deficit in the integration of these sensory cues along the subcortical and/or cortical areas has also been related to balance performance [5,6].

Together with these sensory integration and processing impairments, fatigue is a very frequent complaint (>70%) that contributes to poor balance control, particularly in the most challenging tasks [7]. Furthermore, as postulated by Hebert et al. [8], fatigue can be considered as a significant predictor of balance problems in people with multiple sclerosis. Conversely, although the mechanisms of fatigue related to multiple sclerosis are not completely understood [9], it is reasonable to think that postural instability and difficulty in coordinating eyes, head and body movements during the performance of tasks could also influence fatigue.

The vestibular system has an important role in postural control. In people with multiple sclerosis, several areas along the peripheral and central vestibular pathways may be affected, including the eighth nerve, the vestibular nuclei, the oculomotor tracts, the medial longitudinal fasciculus and the cerebellum [10]. Although central demyelination is expected, peripheral aetiology in vestibular disorders is also very common in multiple sclerosis patients [11,12].

Vestibular rehabilitation consists of exercises that train the sensory systems to provide the correct spatial cues for position as well as for head and body motion [13]. A vestibular physical therapy program may include exercises designed to improve vestibulo-ocular reflex, cervico-ocular reflex, somatosensory retraining, balance and gait [14,15]. It is based on vestibular adaptation and substitution mechanisms [16], which seem to be useful for peripheral and central vestibular lesions [17,18].

Although it is recommended that vestibular rehabilitation is considered for people with multiple sclerosis [19], the benefits of this intervention have not been sufficiently investigated. For this reason, the major purposes of this research were (1) to systematically examine the available evidence of vestibular training interventions for balance and dizziness rehabilitation in multiple sclerosis patients and (2) to determine the magnitude of the effects of these interventions in a meta-analysis.

## 2. Methods

### 2.1. Data Sources and Search Strategy

This systematic review was performed in accordance with the Preferred Reporting Items for Systematic Reviews and Meta-Analyses (PRISMA) guidelines [20]. The review protocol was registered in the PROSPERO database (Registration Number: CRD42019134230).

An electronic search was conducted by two independent reviewers on the following databases: MEDLINE (Pubmed), Scopus, the Physiotherapy Evidence Database (PEDro), Web of Science (WOS), Lilacs, CINHAL and the Cochrane Database of Systematic Reviews (CDSR).

Key words relating to vestibular intervention, outcomes measures and the medical condition under study were combined. Simple or advanced search was conducted when possible. There was no date restriction in any database. All analyses were performed on published data, and thus no ethical approval and patient consent were required.

The search strategy in PubMed was: (“vestibular rehabilitation”[AllFields] OR “vestibular training”[AllFields] OR “vestibular cues”[AllFields] OR “vestibular therapy”[AllFields]) AND ((“posture”[MeSHTerms] OR “posture”[AllFields]) OR (“balance”[AllFields]) OR “postural control”[AllFields] OR imbalance[AllFields] OR (“vertigo”[MeSHTerms] OR “vertigo”[AllFields] OR “dizziness”[AllFields] OR “dizziness”[MeSHTerms]) OR (“vertigo”[MeSHTerms] OR “vertigo”[AllFields])) AND ((“fatigue”[MeSHTerms] OR “fatigue”[AllFields]) OR (“walking”[MeSHTerms] OR “walking”[AllFields]) OR (“gait”[MeSHTerms] OR “gait”[AllFields])) AND “multiple sclerosis”[AllFields]. The search strategy is detailed in Appendix A.

### 2.2. Research Question and Study Selection Criteria

The research question was established following recommendations from the PICOS model (participants, interventions, comparisons, outcomes and study design) as follows: In PwMS, does vestibular rehabilitation improve balance and dizziness symptoms more than other exercise interventions or no intervention?

Thus, patients included in the studies were male and female subjects, clinically diagnosed with multiple sclerosis in accordance with the revised McDonald criteria [21], with walking ability according to the Expanded Disability Status Scale score (EDSS ≤7) and with the objective presence of balance impairment and/or dizziness symptoms.

The intervention was based on the specific vestibular exercise rehabilitation as defined by Whitney et al. [14,15], compared with other general exercise programmes or no intervention. The primary outcome measures were balance and dizziness. Secondary outcomes were fatigue, walking speed and depression.

Randomised controlled trials regarding the effect of vestibular rehabilitation on improving balance and/or dizziness in patients with multiple sclerosis were included. Full texts in English were included. Other methodological designs were excluded.

### 2.3. Data Extraction and Quality Assessment

Two independent reviewers (CGM and MJCH) identified randomised clinical trials from the databases by title and abstract, and any duplicates were removed. Afterwards, a complete reading of the articles was carried out. The reviewers checked that the selected studies met the inclusion criteria.

Once all suitable trials had been selected, the same reviewers performed data extraction independently. Data extraction consisted of collecting information for a qualitative and quantitative synthesis. The main characteristics of the studies (design, participants’ characteristics and sample size, comparison intervention, outcome measures and results) were recorded for the qualitative synthesis, and statistical data were collected for the quantitative synthesis. If the data in the publication were incomplete, the corresponding author was contacted.

Next, these reviewers implemented an independent quality assessment using the Physiotherapy Evidence Database (PEDro) Scale. This scale is based on the Delphi list and is an accepted tool for rating the inner validity of clinical trials [22]. Thus, a study with a score of more than 6 points is considered to be level I evidence (6–8: good; 8–10: excellent). If a randomised controlled trial has a score below 5, it is level II evidence (4–5: deficient; <4: poor). In addition, risk of bias was assessed with the Cochrane Risk of Bias Tool for Randomised Trials [23]. Any disagreements on data extraction or quality assessment were resolved by consensus.

### 2.4. Data Analysis

Results from comparable trials based on intervention parameters, control group and studied outcomes were pooled in a meta-analysis. Mean differences (MD) or standard mean differences (SMD) were calculated with 95% confidence intervals (95% CI). A random-effects model was applied to present the pooled effect. Review Manager (RevMan) (Computer program) Version 5.3. Copenhagen: The Nordic Cochrane Centre, The Cochrane Collaboration, 2014, was used to analyse the effects and construct forest plots and a risk of bias summary.

## 3. Results

### 3.1. Study Selection and Methodological Quality Assessment

**Figure 1:**
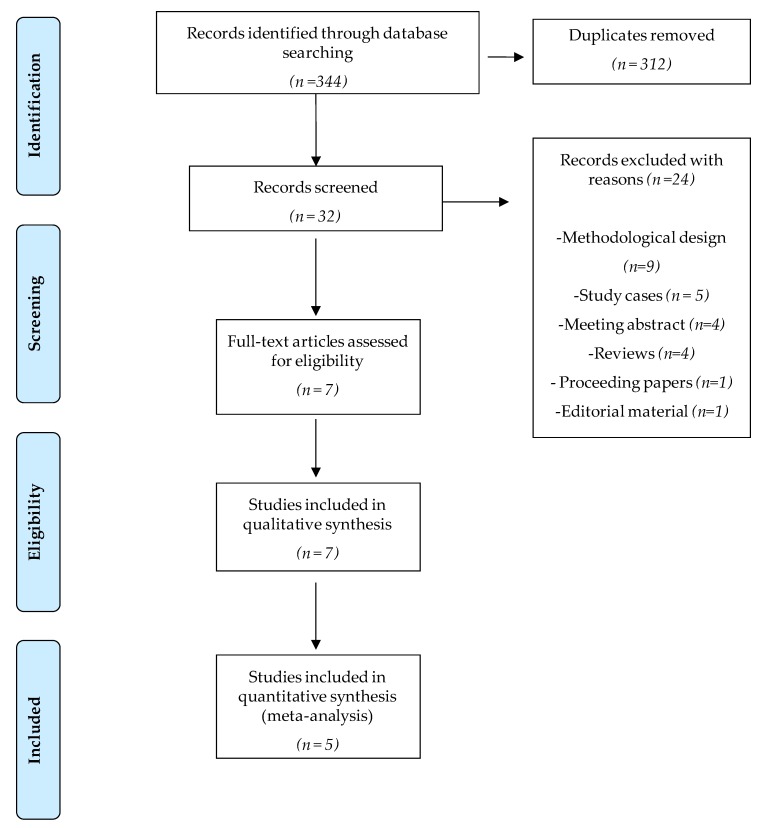
Flow diagram of trial selection based on PRISMA guidelines.

A total of 344 potential papers were identified through the initial database search. After screening, seven papers that met the inclusion criteria were included in this systematic review [24,25,26,27,28,29,30]. They reported results from six randomised controlled clinical trials. All of them were included in the qualitative synthesis, and five were included in the meta-analysis [24,25,27,28,30]. The selection procedure is detailed in the PRISMA methodology flow diagram (Figure 1).

**Figure 2:**
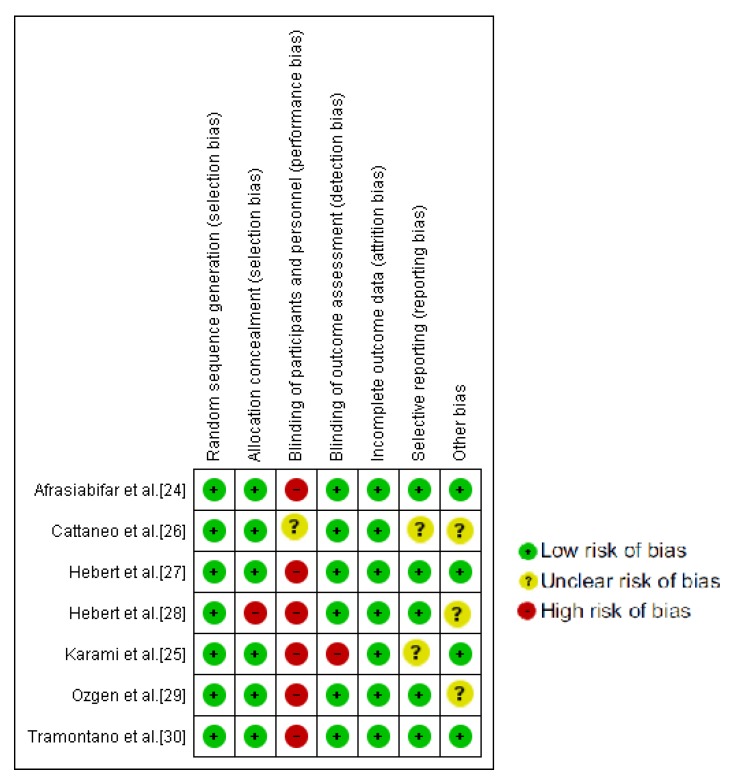
Cochrane risk of bias tool summary.

The PEDro Scale scores ranged from six to eight, with four of the studies being classified as excellent [24,25,27,30]. The blinding of therapists and participants was the most commonly absent item among the PEDro scale criteria. All the studies reported blinding assessors and four of them reported an intention to treat analysis [24,25,27,30]. A complete description of the PEDro Scale can be found in Table 1. As it can be observed in Figure 2, no single study showed a low risk of bias on all domains. Yet, all assessed studies had an acceptable methodological quality.

### 3.2. Study Design and Population Characteristics

The total sample had 321 participants who had been diagnosed with multiple sclerosis (276 women and 93 men, mean age 43.6 years old). Two of the seven studies included compared vestibular rehabilitation with no intervention [28,29] and two of them with other exercise programmes [26,30]. Moreover, three of the studies compared vestibular rehabilitation with both no intervention and other exercise programmes [24,25,27].

In general, the vestibular rehabilitation combined vestibular training plus balance exercises. The vestibular training consisted of head/eyes movement for a task in different positions and on different surfaces with open or closed eyes [26,29]. Two studies made use of the Cawthorne–Cooksey protocol [24,25]. Furthermore, Hebert et al. [28] developed their own vestibular intervention called Balance and Eye-Movement Exercises for Persons with Multiple Sclerosis (BEEMS). The active control group performed exercises based on endurance, strengthening, neuro-rehabilitation exercises, stretching, postural exercises and the Frenkel exercises [24,25]. The main characteristics of the studies included, and some additional information, are shown in Table 2.

### 3.3. Results for Primary Outcomes

#### 3.3.1. Balance

**Figure 3:**
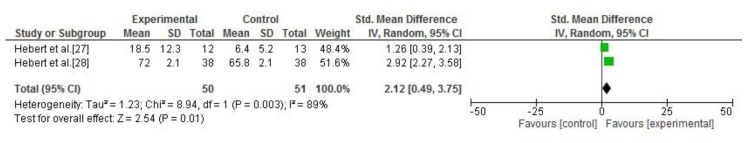
Forest plot of the meta-analysis of postural control (vestibular rehabilitation versus no intervention).

Six of the seven studies analysed postural control or balance ability. This outcome was assessed by posturography [26,27,28,29] and the Berg Balance Scale [24,29,30]. Compared with no intervention, vestibular rehabilitation was more effective for postural control improvements in an upright position (SMD = 2.12; 95% CI = 0.49, 3.75; *p* = 0.01; I^2^ = 89%) [27,28] (Figure 3). Ozgen et al. [29] also compared vestibular rehabilitation versus the usual care for balance training; after intervention, the experimental group obtained greater improvements than the control group, and there were also statistically significant differences between the two groups for the Tandem Romberg and foam standing tests (*p*<0.05). The data from this study were not pooled because the parameters of the intervention were not considered to be sufficiently homogeneous.

**Figure 4:**
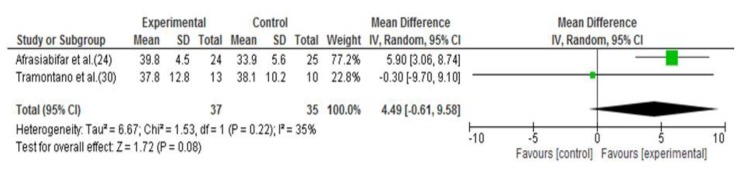
Forest plot of the meta-analysis of Berg Balance Scale (vestibular rehabilitation versus other exercises).

Comparing vestibular therapy to other exercise interventions, the meta-analysis of two studies [24,30] did not report significant differences between the groups (SMD = 4.49; 95% CI = −0.61, 9.58; *p* = 0.08; I^2^ = 35%) [24,30] (Figure 4). Furthermore, Cattaneo et al. [26] also compared vestibular rehabilitation with a standard exercise programme. In this case, statistically significant differences were observed between groups in favour of the experimental intervention for upright postural control in four conditions: eyes closed and firm surface (*p* = 0.033), eyes open and compliant surface (*p* = 0.01), eyes closed and compliant surface (*p* = 0.039) and sway referenced and compliant surface (*p* = 0.017). However, it was not possible to pool the data from this study.

#### 3.3.2. Dizziness

**Figure 5:**
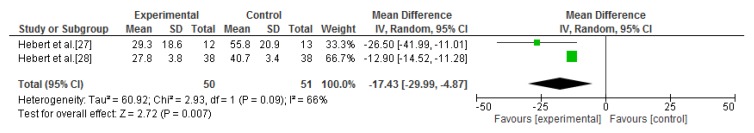
Forest plot of the meta-analysis of dizziness (vestibular rehabilitation versus no intervention).

The Dizziness Handicap Inventory was assessed in three studies [27,28,29]. A meta-analysis was performed, comparing the vestibular rehabilitation and the no-intervention groups. Significant improvements were reported for dizziness in the intervention group (SMD = −17.43; 95% CI = −29.99, −4.87; *p* = 0.007; I^2^ = 66%) [27,28] (Figure 5). Ozgen et al. [29] also assessed the Dizziness Handicap Inventory, but this study was not pooled because its intervention parameters were very different. Intra-group significant differences were only found for the experimental group, but, despite this, there were no statistical differences between the groups.

### 3.4. Results for Secondary Outcomes

#### 3.4.1. Fatigue

**Figure 6:**
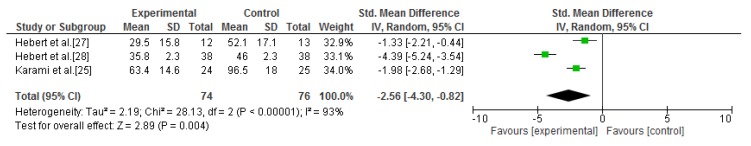
Forest plot of the meta-analysis of fatigue (vestibular rehabilitation versus no intervention).

Four of the seven studies included in this review analysed this outcome [25,27,28,30]. Two different data pools were possible for the meta-analysis of fatigue. The first comparison was between vestibular rehabilitation and no intervention. Three studies could be included in the standardised quantitative analysis [25,27,28]. This analysis showed that vestibular rehabilitation is more effective than no intervention for fatigue improvements (SMD = −2.56; 95% CI = −4.30, −0.82; *p* = 0.004; I^2^ = 93%) [25,27,28] (Figure 6).

**Figure 7:**
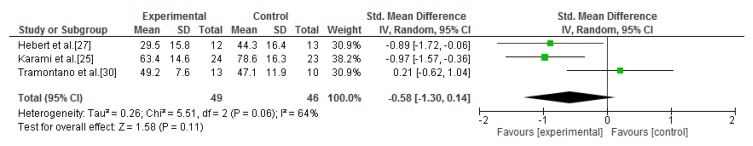
Forest plot of the meta-analysis of fatigue (vestibular rehabilitation versus other exercises).

The second comparison was between vestibular rehabilitation and other general exercise interventions. A standardised quantitative process was carried out including three studies [25,27,30]. Compared with other exercise interventions, improvements in fatigue in the experimental group were observed, but statistical significance was not reached (SMD = −0.58; 95% CI = −1.3, 0.14; *p* = 0.11; I^2^ = 64%) [25,27,30] (Figure 7).

#### 3.4.2. Walking Speed

This outcome was evaluated in three of the seven studies included in this systematic review [28,29,30]. The results were heterogeneous when vestibular rehabilitation was compared with no intervention. Ozgen et al. [29] found statistical intra-group differences only in the experimental group, and significant between-groups differences were also observed. In contrast, Hebert et al. [28] reported no significant between-groups differences. When vestibular exercise was compared with other general exercise programmes, Tramontano et al. [30] observed that participants who underwent vestibular rehabilitation obtained better results than the exercise group, with a statistically significant difference being found between these interventions.

#### 3.4.3. Depression

Depression was assessed in two studies [27,29] by applying the Beck Depression Inventory. There was agreement between the two studies that greater improvements in depressive symptoms were observed in the experimental group, but there were no significant differences between the groups after the intervention. Furthermore, Hebert et al. [27] only observed significant intra-group differences in the experimental group. This study also compared vestibular rehabilitation with the exercise control group. In this case, both interventions gave significant differences after six weeks of training, but no significant differences between the groups were found.

## 4. Discussion

The aim of this study was to summarise and analyse the clinical effectiveness for the improvement of balance and dizziness in people with multiple sclerosis of vestibular rehabilitation in comparison with other physical interventions or no intervention. A total of seven papers reporting six randomised clinical trials were included [24,25,26,27,28,29,30]. Data were pooled from these studies to allow the meta-analysis of three outcomes of interest: balance/postural control, dizziness and fatigue [24,25,27,28,30]. A total of 321 participants with a diagnosis of multiple sclerosis were analysed. Moderate to low risk of bias was observed, except for performance bias.

This meta-analysis supports the argument that vestibular rehabilitation is more effective than no intervention for obtaining improvements in balance, dizziness and fatigue in people with multiple sclerosis. Compared with other physical exercise interventions, improvements among the experimental group were observed, but statistical significance for the differences between groups was not reached. Thus, it can be concluded that vestibular rehabilitation is as effective as other physical therapy and exercise-based interventions. However, these results should be interpreted with caution because of the small number of studies included in the statistical analysis.

Our results for vestibular exercise training are in agreement with the effectiveness of other exercise modalities for balance, gait or fatigue improvements in people with multiple sclerosis reported by the literature [31,32,33]. In a previous systematic review, Palmataa et al. [31] studied the effectiveness of different physical therapy interventions on balance in people with multiple sclerosis. As we do, they concluded that a specific exercise intervention can improve balance ability in people with multiple sclerosis.Similarly, Campbell et al. [32] assessed the efficacy of physical therapy interventions, including exercise therapy, for the rehabilitation of people with progressive multiple sclerosis. They concluded that physical exercise therapy based on endurance, resistance and functional exercises improved mobility, fatigue and depression. In an updated systematic review examining the impact of physical training on disability outcomes, improvements in gait skills after endurance training were also registered.However, as in our study, the general evidence in these systematic reviews was inconclusive because of the small number of studies included.

In comparison with the effectiveness of vestibular rehabilitation in other medical conditions with a deterioration of the vestibular system similar to multiple sclerosis, our results are in accordance with those of a previous review in the elderly [34]. As in the current study, this previous research reported that after a vestibular rehabilitation programme (the Cawthorne–Cooksey protocol was the most commonly employed intervention protocol), dizziness improved in the experimental group. In the elderly population, a progressive loss of nerve cells in the peripheral and central vestibular system, inducing dizziness and vertigo, occurs in a similar way to the occurrence in multiple sclerosis [34].

Similarly, vestibular impairment with persistent symptoms is well known to occur after concussion; as with multiple sclerosis, peripheral and central aetiology is expected [35]. Moreover, similar relationships between fatigue, dizziness and balance performance have been observed in multiple sclerosis [2,7,8] and concussion patients [36]. Previous research in this patient group has concluded that vestibular rehabilitation is an effective and emerging therapy for managing dizziness, vertigo and imbalance [35,37]. In all cases, there was a conclusion that there was a need for more research related to vestibular rehabilitation, as is reported in this study.

### 4.1. Study Limitations

This review presents some limitations. First, the number of studies included is small, although only randomised controlled trials were included and the methodological quality was moderate to high. Second, heterogeneity in vestibular rehabilitation interventions was observed. Although only data from reasonably homogeneous interventions were pooled, it is difficult to generalise the results. This limitation is frequently reported in other systematic reviews regarding vestibular rehabilitation interventions [34,35]. Third, different types of multiple sclerosis were included; however, as in the present review, it is frequent for non-pharmacological interventions to establish inclusion criteria based on disability related to multiple sclerosis instead of the subtype of the disease [38,39]. Fourth, only two studies analysed the presence of brainstem/cerebellar involvement based on magnetic resonance imaging scans and neurologic examinations [27,28], and only one of them contrasted the results taking this condition into account [28]. Although vestibular rehabilitation repair mechanisms are suitable for both peripheral and central lesions [18], and several areas of lesions are expected in multiple sclerosis, the clinical confirmation of these lesion sites could be of interest.

### 4.2. Clinical Implications

On the other hand, this study also has some clinical implications for rehabilitation practice. First, it is the first systematic review and meta-analysis based on randomised controlled trials to be carried out with the aim of studying the effectiveness of vestibular rehabilitation in multiple sclerosis. As Dunlap et al. [40] assert, there was a gap in the knowledge about the topic in this population. Second, the current review concludes that this intervention is at least as effective as other exercise-based interventions and more effective than no intervention. Furthermore, vestibular rehabilitation appears to be an easy and affordable intervention [16,41], with adherence in home programmes if therapists provide summaries to patients of the intervention [42].

To improve the strength of the evidence, although vestibular rehabilitation has been suggested as an effective intervention in multiple sclerosis, more research supporting this conclusion is needed. Furthermore, outcomes like quality of life or functionality may be included in future studies. These studies need to report on clear protocols and comparable interventions between groups.

## Figures and Tables

**Table 1:** PEDroscale items assessment.

Section/Theme	Afrasiabifar et al. [24]	Karami et al. [25]	Cattaneo et al. [26]	Hebert et al. [27]	Hebert et al. [28]	Ozgen et al. [29]	Tramontano et al. [30]
Eligibility criteria	Yes	Yes	Yes	Yes	Yes	Yes	Yes
Random allocation	Yes	Yes	Yes	Yes	Yes	Yes	Yes
Concealled allocation	Yes	Yes	Yes	Yes	No	Yes	Yes
Baseline comparability	Yes	Yes	Yes	Yes	Yes	Yes	Yes
Blind subjects	No	No	Yes	No	No	No	No
Blind therapists	No	No	No	No	No	No	No
Blind assessors	Yes	Yes	Yes	Yes	Yes	Yes	Yes
Adequate follow-up	Yes	Yes	Yes	Yes	Yes	Yes	Yes
Intention to treat analysis	Yes	Yes	No	Yes	No	No	Yes
Between-group comparisons	Yes	Yes	Yes	Yes	Yes	Yes	Yes
Point estimates and variability	Yes	Yes	No	Yes	Yes	Yes	Yes
Total score	8/10	8/10	7/10	8/10	6/10	7/10	8/10

**Table 2:** Main characteristics of the studies included.

Study	Design, PEDro Score	Participants, Characteristics and Sample Size	Intervention (VR Group)	Control Group	Outcome Measures	Main Results
**Afrasiabifar et al. [24]**	RCT, 8/10	EG mean age(SD): 32.4 (8.1) G2 mean age SD): 32 (6.7) CG mean age (SD): 33.6 (7.3) N = 72 (EG: 24, G2: 23, CG: 25), 3 drop out 64 MSRR 4 MSPP/SP Location of intervention: clinic	EG: Cawthorne–Cooksey vestibular rehabilitation exercise protocol 3 session week, 60 min 12 weeks intervention	G2: Frenkel exercises, 3 session per week, 60 min CG: no intervention	Berg Balance Scale (BBS)	EG-CG/G2: Significant differences in favour of the experimental group for BBS in comparison with CG (*p* = 0.001) and G2 (*p* = 0.001) G2-CG: Significant differences in favour of G2 for BBS (*p* = 0.01)
**Karami et al. [25]**	RCT, 8/10				Fatigue: FIS	EG-CG/G2: Significant differences in favour of the experimental group in comparison with the CG (*p* = 0.001) and G2 (*p*= 0.007) G2-CG: Significant differences in favour ofthe G2 (*p* = 0.001)
**Cattaneo et al. [26]**	RCT 7/10	EG mean age(SD): 48.5 (11.01) CG mean age (SD): 48.2 (12.05) N = 53 (EG: 25, CG: 28) 24 MSRR 3 MSPP 26 MSSP EDSS: 1–6.5 Location: clinic hospital.	EG: Balance exercises with open/closed eyes on different surfaces plus head–eyes movements (vestibular cues) 15 sessions of 45 min, 3 weeks intervention	CG: usualrehabilitation based on range motion, muscle force, postural changes and gait on firm surface 15 sessions of 45 min, 3 weeks intervention	Balance: COP disturbances in six different conditions (eyes open/closed and sway referenced on firm and foam surfaces)	EG-CG: Significant differences in favour of the experimental group for eyes closed firm surface (*p* = 0.033, eyes opencompliant surface (*p* = 0.01), eyes closed compliant surface (*p* = 0.039) and sway referenced compliant surface (*p* = 0.017)
**Hebert et al. [27]**	RCT, 8/10	EG mean age(SD): 46.8 (10.5) G2 mean age(SD): 42.6 (10.4) CG mean age (SD): 50.2 (9.2) N = 38 (EG: 12, G2: 13, CG: 13) 34 MSRR 4MSSP Location: human performance laboratory and home exercises	EG: upright postural control and eye movement exercises, Perform of 1–2 min each item 2 session/week, 60 min + a daily independent home exercise program 6 weeks of intervention	G2: endurance of static bicycling and stretching exercise of lower limb muscles; stretches were held for 30 s Samefrequency and duration CG: no intervention	Balance: posturography (SOT) Fatigue: MFIS Dizziness: DHI Exercise capacity: 6MWT Depression: BDI-II	EG-CG/G2: Significant differences in favour of the experimental group in comparison with control group for balance (*p*=0.003), fatigue (*p*=0.005) and dizziness (*p*=0.009) and G2 for balance(*p*< 0.001) G2-CG: No statistical differences between groups for any of the variables
**Hebert et al. [28]**	RCT, 6/10	EG Mean age(SD): 46.5 (8.8) CG Mean age(SD): 43 (10.8) N = 88 (EG: 44, CG: 44), 12 drop out EDSS: 3.34–3.5 Location: human performance laboratoryand home exercises	EG: BEEMS protocol, 2 s/w + daily home exercise (phase 1). Phase 2: 1 s/w + daily home exercises 14 weeks intervention	CG: no intervention	Balance: CDP-SOT Fatigue: MFIS Dizziness: DHI, DVAT, GST Gait: T25FW Quality of life: PDQ, SF-36 Mental and Physical component	EG-CG: Significant differences in favour of the experimental group for balance, fatigue and dizziness/DHI (*p*< 0.0001); quality of life/PDQ (*p*=0.0008), SF-36 MC (*p* = 0.02) and SF-36 PC (*p* = 0.01) A greater improvement was observedin participants with brainstem/cerebellar lesion compared with those without in CDP-SOT composite (*p* = 0.04) and MFIS total (*p* = 0.02)
**Ozgen et al. [29]**	RCT, 7/10	EG mean age: 42.5 CG mean age: 39.5 N = 40 (EG: 20, CG: 20) 17MSRR 7MSPP 16MSSP Location: human performance laboratory	EG: vestibular rehabilitation program (balance and ambulation exercises) 15–20 min, twice a day 8 weeks of intervention	CG: no intervention	Balance: static posturography, BBS, Romberg tests + foam, FTSTS, TUG, ABC Dizziness: DHI Gait: 6WT, DGI, FGA, Depression: BDI Qualitylife: MSQoL-54	EG-CG: Significant differences between groups for Tandem Romberg (eyes closed) and foam standing (eyes open) (*p*<0.05)
**Tramontano et al. [30]**	RCT, 8/10	EG mean age (SD): 50.64 (11.73) CG Mean age: 45.77 (10.91) N = 30 (EG: 15, CG: 15) 7 dropout EDSS: 6–7 Location: clinic	EG: conventional neurorehabilitation therapy for MS + 10 min exercise for gaze stability + 10 exercise for postural stability Two daily 40 min + 20 min VR, 5days/week 4 weeks intervention	CG: two daily 40-min 5 days/w for 4 weeks of conventional neurorehabilitation therapy for MS. Muscle stretching, postural alignment, active-assisted mobilisations and neuromuscular facilitations, balance training: progressive restrictions of support base, unstable surfaces.	Balance: TBG, BBS Fatigue: FSS Gait: 2MWT, T25FW Disability: EDSS, BI	EG-CG: Significant differences in favour of the experimental group were observed except for 2MWT

**2MWT:** Two Minute Walking Test; **6MT:** Six meter walk test; **6MWT:** Six-Minute Walk Test; **ABC:** Activities-Specific Balance Confidence; **BBS:** Berg Balance Scale; **BDI-II:** Beck Depression Inventory–II; **BEEMS:** Balance and Eye-Movement Exercises for Persons with MultipleSclerosis; **BI:** Barthel Index. **CDP-SOT:** Computerised Dynamic Posturography-Sensory Organisation Test; **CG:** control group; **COP**: Center of pressure; **DGI:** Dynamic Gait Index; **DHI:** Dizziness Handicap Inventory; **DVAT:** Dynamic Visual Acuity Test; **EDSS:** Expanded Disability Status Scale; **EG:** experimental group; **FGA:** Functional Gait Assessment; **FIS:** Fatigue Impact Scale; **FTSTS:** Five Times Sit-to-Stand Test; **FSS:** Fatigue Severity Scale; **G2:** secondary intervention group; **GST:** Gaze Stabilisation Test; **MFIS:** Modified Fatigue Impact Scale; **min:** minute; **MS:** multiple sclerosis; **MSRR:** Multiple Sclerosis relapsing remitting; **MSPP:** Multiple sclerosis primary progressive; **MSSP:** Multiple sclerosis secondary progressive; **MSQoL-54:** Multiple Sclerosis Quality of Life Scale–54; **PDQ:** Perceived Deficits Questionnaire; **RCT:** randomised clinical trial; **s/w:** session/week; **SD:** standard deviation; **SF-36:** Short Form-36 Health Status Questionnaire; **SOT:** SensoryOrganisation Test (posturography); **T25FW:** Timed 25-foot walk test; **TBG:** Tinetti Balance Gait scale; **TUG:**Timed Up-and-Go Test; **VAS:** Visual Analog Scale; **VR:** Vestibular rehabilitation.

## Appendix A. Detailed Search Strategy

**Nº term used**
**#1** “Vestibular rehabilitation” **#2** “Vestibular training” **#3** “Vestibular cues” **#4** “Vestibular therapy” **#5** “Multiple sclerosis” **#6** “Fatigue” **#7** “Posture” **#8** “Balance” **#9** “Postural control” **#10** “Imbalance” **#11** “Dizziness” **#12** “Vertigo” **#13** “Walking” **#14** “Gait”

**PUBMED (6 potential articles):**

(“vestibular rehabilitation”[AllFields] OR “vestibular training”[AllFields] OR “vestibular cues”[AllFields] OR “vestibular therapy”[AllFields]) AND ((“posture”[MeSHTerms] OR “posture”[AllFields]) OR (“balance”[AllFields]) OR “postural control”[AllFields] OR imbalance[AllFields] OR (“vertigo”[MeSHTerms] OR “vertigo”[AllFields] OR “dizziness”[AllFields] OR “dizziness”[MeSHTerms]) OR (“vertigo”[MeSHTerms] OR “vertigo”[AllFields])) AND ((“fatigue”[MeSHTerms] OR “fatigue”[AllFields]) OR (“walking”[MeSHTerms] OR “walking”[AllFields]) OR (“gait”[MeSHTerms] OR “gait”[AllFields])) AND “multiple sclerosis”[AllFields]

**Web of Science (115 potential articles):**

- #1 OR #2 OR #3 OR #4 AND #7 OR #8 OR #9 OR #10 OR #11 OR #12 AND #6 OR #13 OR #14 AND #5 (6 potential articles)
- #1 OR #2 OR #3 OR #4 AND #7 OR #8 OR #9 OR #10 OR #11 OR #12 AND #5 (26 potential articles)
- #1 OR #2 OR #3 OR #4 AND #6 OR #13 OR #14 AND #5 (16 potential articles)
- #1 OR #2 OR #3 OR #4 AND #6 AND #5 (11 potential articles)
- #1 OR #2 OR #3 OR #4 AND #13 OR #14 AND #5 (11 potential articles)
- #1 OR #2 OR #3 OR #4 AND #11 OR #12 AND #5 (17 potential articles)
- #1 OR #2 OR #3 OR #4 AND #5 (28 potential articles)

**SCOPUS (83 potential articles):**

- #1 OR #2 OR #3 OR #4 AND #7 OR #8 OR #9 OR #10 OR #11 OR #12 AND #6 OR #13 OR #14 AND #5 (10 potential articles)
- #1 OR #2 OR #3 OR #4 AND #7 OR #8 OR #9 OR #10 OR #11 OR #12 AND #5 (19 potential articles)
- #1 OR #2 OR #3 OR #4 AND #6 OR #13 OR #14 AND #5 (10 potential articles)
- #1 OR #2 OR #3 OR #4 AND #6 AND #5 (4 potential articles)
- #1 OR #2 OR #3 OR #4 AND #13 OR #14 AND #5 (9 potential articles)
- #1 OR #2 OR #3 OR #4 AND #11 OR #12 AND #5 (12 potential articles)
- #1 OR #2 OR #3 OR #4 AND #5 (19 potential articles)

**Cochrane Database of Systematic Reviews (73 potential articles):**

- #1 OR #2 OR #3 OR #4 AND #7 OR #8 OR #9 OR #10 OR #11 OR #12 AND #6 OR #13 OR #14 AND #5 (10 potential articles)
- #1 OR #2 OR #3 OR #4 AND #7 OR #8 OR #9 OR #10 OR #11 OR #12 AND #5 (14 potential articles)
- #1 OR #2 OR #3 OR #4 AND #6 OR #13 OR #14 AND #5 (11 potential articles)
- #1 OR #2 OR #3 OR #4 AND #6 AND #5 (8 potential articles)
- #1 OR #2 OR #3 OR #4 AND #13 OR #14 AND #5 (9 potential articles)
- #1 OR #2 OR #3 OR #4 AND #11 OR #12 AND #5 (5 potential articles)
- #1 OR #2 OR #3 OR #4 AND #5 (16 potential articles)

**PEDro (8 potential articles):**

- #1 AND #5 (4 potential articles)
- #1 AND #8 AND #5 (3 potential articles)
- #1 AND #6 AND #5 (2 potential articles)
- #1 AND #11 AND #5 (2 potential articles)
- #1 AND #14 AND #5 (1 potential article)

**LILACS (6 potential articles):**

- #1 AND #5 (2 potential articles)
- #1 AND #8 AND #5 (1 potential article)
- #1 AND #11 AND #5 (2 potential articles)
- #1 AND #14 AND #5 (1 potential article)

**CINAHL (53 potential articles)**:

- #1 OR #2 OR #3 OR #4 AND #7 OR #8 OR #9 OR #10 OR #11 OR #12 AND #6 OR #13 OR #14 AND #5 (5 potential articles)
- #1 OR #2 OR #3 OR #4 AND #7 OR #8 OR #9 OR #10 OR #11 OR #12 AND #5 (19 potential articles)
- #1 OR #2 OR #3 OR #4 AND #6 OR #13 OR #14 AND #5 (5 potential articles)
- #1 OR #2 OR #3 OR #4 AND #6 AND #5 (5 potential articles)
- #1 OR #2 OR #3 OR #4 AND #13 OR #14 AND #5 (3 potential articles)
- #1 OR #2 OR #3 OR #4 AND #11 OR #12 AND #5 (7 potential articles)
- #1 OR #2 OR #3 OR #4 AND #5 (9 potential articles)

Note: only strategies with results are showed.
